# High-Resolution Ultrasound-Assisted Assessment of Preliminary Short-term Safety Outcomes of an Implant-Based Augmentation Mammaplasty Using a Bioengineered, Cell-Friendly, Smooth-Surface Device in Korean Females

**DOI:** 10.1093/asjof/ojab046

**Published:** 2021-11-09

**Authors:** Sangdal Lee, Jeong Pil Jeong, Jung Youp Sung, Woo Sik Choi, Dong Seung Moon, Ho Chan Kim, Jae Hong Kim

## Abstract

**Background:**

The Motiva Ergonomix Round SilkSurface (Establishment Labs Holdings Inc., Alajuela, Costa Rica) is one of the representative brands of the fifth generation of a silicone gel-filled breast implant with a microtextured surface.

**Objectives:**

In this study, the authors describe preliminary short-term safety outcomes of an implant-based augmentation mammaplasty using the Motiva Ergonomix Round SilkSurface in Korean females.

**Methods:**

The authors performed a retrospective analysis of medical records in a total of 69 females (n = 69) receiving an implant-based augmentation mammaplasty using the Motiva Ergonomix Round SilkSurface between September 26, 2017, and December 31, 2020. The authors analyzed incidences of postoperative complications.

**Results:**

A total of 6 cases (8.7%) of postoperative complications occurred; these include 2 cases (2.9%) of early seroma, 1 case (1.4%) of capsular contracture, 2 cases (2.9%) of alterations in the shape, and 1 case (1.4%) of foreign body sensation. Time-to-events were estimated at 266.81 ± 273.17 days.

**Conclusions:**

The authors describe our preliminary short-term safety outcomes of an implant-based augmentation mammaplasty using the Motiva Ergonomix Round SilkSurface in Korean females. But this deserves further large-scale studies with long periods of follow-up.

**Level of Evidence: 4:**

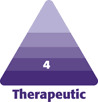

The use of a silicone gel-filled breast implant has been approved for augmentation mammaplasty, thus termed as an implant-based augmentation mammaplasty, by the US Food and Drug Administration (FDA) in females. Its indications include aesthetic breast enlargement, post-mastectomy reconstruction, or correction of developmental defects or outcomes of a previous surgery.^1,2^ Moreover, a silicone gel-filled breast implant is characterized by a variability in its shell, gel, surface topography, and shape.^3^ To date, plastic surgeons, manufacturers of a silicone gel-filled breast implant, and patients have experienced a transition from one device to another worldwide.^2,4^

Extensive testing procedures are required to make a silicone gel-filled breast implant commercially available in the market; its efficacy and safety should be stringently assessed. Nevertheless, patients receiving an implant-based augmentation mammaplasty remain at risk of developing postoperative complications.^5,6^ These include additional surgeries, capsular contracture (CC), breast implant illness, breast implant-associated anaplastic large cell lymphoma (BIA-ALCL), pain, rupture, or infection.^7-9^

The value of a silicone gel-filled breast implant market is estimated at USD 1.43 billion in 2019 and then expected to reach USD 2.2 billion by 2026.^10^ Korean market of a silicone gel-filled breast implant is characterized by competition between the global key players; these include the Polytech Health and Aesthetics (Dieburg, Germany), the Allergan Inc. (Irvine, CA), Groupe Sebbin SAS (Boissy-l′Aillerie, France), the Mentor Worldwide LLC (Santa Barbara, CA), the Sientra Inc. (Rio de Janeiro, Brazil), the Establishment Labs Holdings Inc. (Alajuela, Costa Rica), and the HansBiomed Co. Ltd. (Seoul, Korea).^11,12^ Moreover, it has also been recently characterized by the popularity of a microtextured device since the Motiva Ergonomix Round SilkSurface (Establishment Labs Holdings Inc.) was approved by the Korean Ministry of Food and Drug Safety (KMFDS) on June 17, 2016.^13,14^ Use of a microtextured breast implant has been accelerated since the clinical use of a macrotextured device was banned by the KMFDS on August 29, 2019, when it announced the first Korean case of BIA-ALCL to the public.^15^

We previously compared 1-year safety outcomes between the 2 representative brands of a microtextured breast implant in Korea: the Motiva Ergonomix Round SilkSurface and the BellaGel SmoothFine.^13^ Interestingly, previous manufacturer-sponsored studies have also compared the safety and vulnerability to CC between the 2 devices.^16-18^ Moreover, 4-year interim results of the safety of the Motiva Ergonomix Round SilkSurface have also been published in a peer-reviewed journal.^19^ Still, however, there is a paucity of literatures showing safety outcomes of an implant-based augmentation mammaplasty using the Motiva Ergonomix Round SilkSurface based on high-resolution ultrasound (HRUS; Aplio i600, Canon Medical System, Otawara, Tochigi, Japan).

Given the above background, we have used HRUS in detecting postoperative complications at the earliest opportunities possible in patients receiving an implant-based augmentation mammaplasty.^15,20^ We, therefore, conducted this study to assess preliminary short-term safety outcomes of the Motiva Ergonomix Round SilkSurface in our clinical series of the patients who had been postoperatively followed up using HRUS.

## METHODS

### Study Patients and Setting

Between September 26, 2017, and December 31, 2020, a total of 119 patients (238 breasts) received an implant-based augmentation mammaplasty using the Motiva Ergonomix Round SilkSurface at our hospitals. We included females aged 18 years or older with a normal physical development receiving the Motiva Ergonomix Round SilkSurface for augmentation mammaplasty. But exclusion criteria include (1) reconstruction (n = 3), (2) revision or reoperation (n = 48), (3) subglandular pocket (n = 5), or (4) loss of follow-up (n = 2). The current study was conducted in compliance with the relevant ethics guidelines; it was approved by the Internal Institutional Review Board of the Korea National Institute of Bioethics Policy (P01-202101-21-021). All the procedures described herein were performed in accordance with the 1964 Declaration of Helsinki and its later amendments or comparable ethical standards. Written consent was provided by which the patients agreed to the use and analysis of their data.

#### The Manufacturer’s Description of the Motiva Ergonomix Round SilkSurface

**Figure 1.**
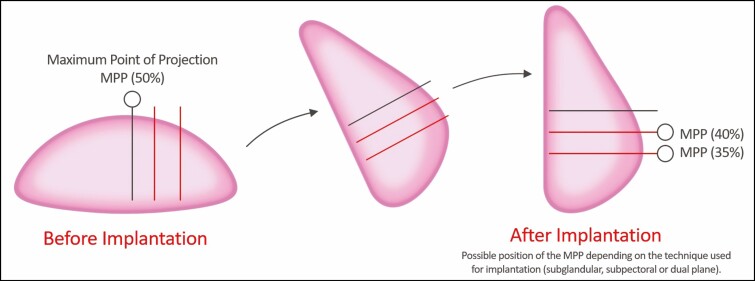
Maximum point of projection (MPP). According to the manufacturer, the Motiva Ergonomix Round SilkSurface is advantageous in shifting the MPP to the lower pole of the breast when patients are in a standing posture and to the middle pole of the breast when they lie flat on their back in a similar manner to a natural breast.

According to the manufacturer, the Motiva Ergonomix Round SilkSurface is defined as a bioengineered, cell-friendly, smooth-surface breast implant; it is the fifth generation of a silicone gel-filled breast implant with a microtextured surface.^21^ Its technological properties are based on the TrueTissue Technology that is a combination of a specific elastic elastomer shell with special rheological properties of the ProgressiveGel Ultima. Thus, the Motiva Ergonomix Round SilkSurface adjusts with gravity to a patient’s posture. With gravity, the maximum point of projection (MPP) shifts to the lower pole of the breast when patients are in a standing posture. The MPP shifts to the middle pole of the breast when they lie flat on their back in a similar manner to a natural breast (Figure 1).^21-24^

The Motiva Ergonomix Round SilkSurface is equipped with a refined, smooth surface with a roughness of 3.18 μm according to the International Organization for Standardization (ISO) 14607 Appendix H Test for surface characteristics. Based on nanotechnology, it is equipped with the smallest surface with 49,000 contact points of 16 μm (16,000 nm) depth per cm.^2,19^

#### Treatment Protocol

We perform an implant-based augmentation mammaplasty in a step-by-step manner, followed by a multi-disciplinary, algorithm-based approach to an early detection of postoperative complications, as previously described.^11,13,15,20^ Moreover, the patients were not only regularly followed up at 1, 2, 3, and 4 weeks; 3 months; and 1 year, but they were also examined, if necessary, at 6 and 9 months postoperatively. 

##### Preoperative Simulation of Postoperative Outcomes

Preoperatively, we use the Divina 3-dimensional Scanner (Establishment Labs Holdings Inc., Alajuela, Costa Rica) to allow the patients to view possible results of an implant-based augmentation mammaplasty. It not only helps a surgeon obtain anthropometric measurements of the breast, such as the width and height of breast base; distance from the sternal notch to the nipple, that from the nipple to the midline, that from the nipple to the inframammary fold (IMF); areolar diameter; internipple distance; intermammary distance; and breast volume, but also visualizes its preoperative characteristics. Thus, it stimulates possible results through an analysis of data and information about diverse types of a silicone gel-filled breast implant for the purposes of helping patient select optimal types of a breast implant and thereby yielding satisfactory outcomes.^20^

##### Surgical Procedures

Our surgical procedures are performed in compliance with the American Society of Plastic Surgeons (ASPS) recommendations. Peri-areolar, IMF, and trans-axillary incisions were made under general anesthesia and intravenous sedation for the purposes of preventing visible scarring. The selection of surgical incision is based on our desired outcomes, types of breast implants, the degree of augmentation, the anatomical characteristics of patients, and patient-surgeon preference. Based on the Ranquist formula, we determined the distance extending from the nipple to the IMF, the size of breast implant, and the scope of dissection. After the dissection, each breast was irrigated using 100 mL of normal saline mixed with H_2_O_2_ solution at a ratio of 1:1, followed by the use of betadine 100 mL. Then, a breast implant was immersed in a normal saline mixed with ceftezole 1 vial and gentamycin 1 ample and then inserted in a pocket either under the *pectoralis* muscle (a submuscular placement) or in the retromammary space above the *pectoralis major* muscle (a subglandular/submammary placement). Methods for inserting and positioning a breast implant in the pocket were dependent on its types, the degree of augmentation, characteristics of a patient’s body, and our recommendations. Thus, we performed a dual-plane I/II augmentation on a case-by-case basis. Intraoperatively, the patients were intravenously given ceftezole 1.0 g. Incisions were closed using layered sutures in the breast tissue. In addition, skin adhesive or surgical tape was used to close the skin.^11,13^

##### HRUS-Assisted Measurement of Capsule Thickness

To make an accurate diagnosis of CC, we measure the capsule thickness at 3 months postoperatively in the patients who are suspected of having CC. Moreover, we consider an empirical correlation between the capsule thickness on HRUS and the Baker classification system (Baker grades I [<0.4 mm], II [0.4-0.8 mm], III [0.8-1.4 mm], and IV [>1.4 mm]). If necessary, we perform capsulectomy and thereby collect tissue samples to make an accurate diagnosis of complications, as described in a recent study.^20,25^

##### Planning of Revisional Surgery Based on HRUS

We plan for revisional surgery considering the capsule thickness and whether the patients present with any notable signs and symptoms when they had an increase in it on HRUS at 3 months postoperatively. If necessary, we frequently perform a follow-up of the corresponding patients to examine whether they present with changes in the capsule thickness and symptoms. Thus, we determine whether they required revisional surgery.^20^

##### HRUS-Assisted Measurement of the Thickness of Dermis, Subcutaneous Tissue, and *Pectoralis Major* Muscle

To examine whether the patients present with swelling after an implant-based augmentation mammaplasty, we measure the thickness of dermis, subcutaneous tissue, and *pectoralis major* on HRUS preoperatively and at 1 and 3 months, postoperatively.^20^

#### Patient Evaluation and Criteria

We analyzed baseline and clinical characteristics of the patients, as previously described.^11,13,15,20^ To analyze the safety of the Motiva Ergonomix Round SilkSurface, we classified postoperative complications into surgery- and implant-related ones. Moreover, we considered risk factors and thereby performed a subgroup analysis of them. Manufacturers’ core studies have shown significantly higher incidences of CC in secondary cases, such as revisional surgery or reoperation, as compared with primary ones.^26-29^ Moreover, lower incidences of CC had a significant correlation with the use of anatomical implants.^30^ Furthermore, locations of the implant pocket also serve as risk factors of developing CC; a subglandular pocket is commonly associated with higher incidences of CC as compared with a submuscular or dual-plane one.^31^ We, therefore, excluded factors such as secondary cases, anatomical devices, and subglandular pocket in analyzing incidences of postoperative complications.

To evaluate survivorship of the patients without complications of an implant-based augmentation mammaplasty, we estimated complication-free survival, as calculated as percentage of the Motiva Ergonomix Round SilkSurface remaining without undergoing revision or removal of it without revision.^11,13,15,19,20^

#### HRUS-Assisted Assessment of CC Based on Thickened Capsule

In assessing the severity of CC, we analyzed thickened capsule (TC), defined as an abnormal thickening of the capsule seen on HRUS irrespective of whether it is total or partial in nature, that serves as an indicator of CC on HRUS.^13,20^ This is based on a rationale that a novel classification system based on the TC should be established for making an objective diagnosis of CC because the Baker classification system is not a reliable diagnostic tool for CC; TC entails the Baker grades II-IV, but CC is restricted to the Baker grades III/IV.^25^

From empirical perspectives, we commonly observe that TC is an objective indicator of CC. That is, a lack of TC would lead to that of CC. It cannot be ensured, however, that the presence of TC would lead to a diagnosis of CC. Moreover, a lack of TC until postoperative 3-6 months commonly leads to that of CC over time. It can, therefore, be inferred that TC might not serve as a risk factor for CC in patients without TC until postoperative 3-6 months. Furthermore, we also commonly encounter patients with CC who had a past history of having TC corresponding to CC of Baker grade II between postoperative 1 and 3 months. This is noteworthy because patients with TC corresponding to CC of Baker grade II would be commonly neglected without an ultrasound-assisted approach. These patients should be meticulously monitored for the possible progression to CC of Baker grade III/IV. But such progression is observed on a case-by-case basis. Indeed, TC is such a reliable indicator of CC as to predict the occurrence of CC.

Both TC and its scope are essential factors for making an accurate diagnosis, treatment, and prognosis of CC. Increased thickness of the capsule at a single site cannot solely serve as a diagnostic clue to CC of Baker grade III/IV; the scope of TC is more important than the capsule thickness itself. It is of no doubt that conventional Baker classification system is also closely associated with the capsule thickness.^25^ It remains problematic, however, that the Baker classification system fails to address the scope of TC. In more detail, TC of Baker grade II should be diagnosed as an abnormal condition considering both the capsule thickness and the scope of TC. We, therefore, use novel diagnostic criteria for CC, termed as the Kim JH (KJH) classification system, alternatively to the Baker classification system, as summarized in Table 1. 

**Table 1.** The KJH Classification System and Its Equivalent Baker Grade

	Diagnostic criteria		Equivalent Baker grades
	TC on HRUS	Symptoms	
KJH grade I	(−)	(−)	Baker grade I
KJH grade II	(+)	(−)	Baker grades I/II
KJH grade III	(+)	(+)	Baker grades III/IV

HRUS, high-resolution ultrasound; KJH, Kim JH; TC, thickened capsule.

#### Statistical Analysis

All data were expressed as mean ± standard deviation (SD) or mean ± standard error (SE), where appropriate. Continuous variables were analyzed using the repeated measures analysis of variance (ANOVA), the Kruskal-Wallis test, or Fisher’s exact test. Non-continuous variables were analyzed using the χ ^2^-test. The cumulative overall complication-free survival was estimated, for which 95% confidence intervals (CIs) were provided. In addition, the corresponding cumulative complication-free Kaplan-Meier survival and hazards were plotted as a curve. Statistical analysis was done using the SPSS ver. 18.0 for windows (SPSS Inc., Chicago, IL). A *P*-value of <0.05 was considered statistically significant.

## RESULTS

### Baseline Characteristics of the Patients

In our series, there were 2 patients receiving reconstructive surgery, corresponding to secondary case, 1 of whom was lost to follow-up. Moreover, there were 4 patients receiving a device in the subglandular pocket, corresponding to secondary case. Therefore, a total of 69 females (n = 69) met inclusion/exclusion criteria, whose mean age was 34.2 ± 8.2 (18-51) years old. They were followed up during a mean period of 292.5 ± 276.4 (1-1151) days. Their baseline characteristics are presented in Table 2. On HRUS, 57, 5, 22, 12, and 4 patients were followed up at 3 and 6 months and 1, 2, and 3 years postoperatively, respectively.

**Table 2.** Baseline Characteristics of the Patients (n = 69)

Variables	Values
Age (years old)	34.2 ± 8.2 (18-51)
Sex (male-to-female ratio)	0:69
Height (cm)	163.5 ± 5.1
Weight (kg)	51.6 ± 5.5
BMI (kg/m^2^)	19.3 ± 1.8
FU period (days)	292.5 ± 276.4 (1-1151)
Purpose of surgery	
Aesthetic augmentation mammaplasty	
Left side	69 (100.0%)
Right side	69 (100.0%)
Round of surgery	
Primary cases	69 (100.0%)
Type of incision	
Trans-axillary incision	68 (98.6%)
Peri-areolar incision	1 (1.4%)
Type of pocket	
Subpectoral pocket	69 (100.0%)
Volume of breast implant	
Left side	
≤245 cc	2 (2.9%)
250-295 cc	29 (42.0%)
300-345 cc	22 (31.9%)
350-395 cc	13 (18.8%)
≥400 cc	3 (4.3%)
Right side	
≤245 cc	1 (1.4%)
250-295 cc	26 (37.7%)
300-345 cc	24 (34.6%)
350-395 cc	9 (13.0%)
≥400 cc	9 (13.0%)
Profile of breast implant	
Left side	
Ultra-high	0 (0.0%)
High	60 (87.0%)
Medium	9 (13.0%)
Low	0 (0.0%)
Non-applicable	0 (0.0%)
Right side	
Ultra-high	0 (0.0%)
High	64 (92.8%)
Medium	5 (7.2%)
Low	0 (0.0%)
Non-applicable	0 (0.0%)

Values are mean ± standard deviation or the number of cases with percentage, where appropriate. BMI, body mass index; FU, follow-up.

### Clinical Case

**Figure 2.**
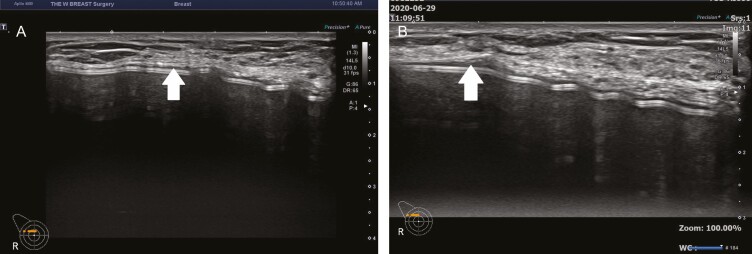
Clinical case. A 51-year-old female received primary augmentation mammaplasty using the Motiva Ergonomi Round SilkSurface (ERSF; 250 cc for both breasts) in a dual-plane I, subpectoral pocket *through* a trans-axillary incision. (A) On postoperative day 90, the patient had a thickened capsule (TC) in the right breast, corresponding to capsular contracture of Baker grade II. (B) On postoperative day 190, however, the patient exhibited no further changes in TC. Arrow indicates TC.

A case of a 51-year-old female with a TC after receiving a device is shown in Figure 2.

### Safety Outcomes

**Figure 3.**
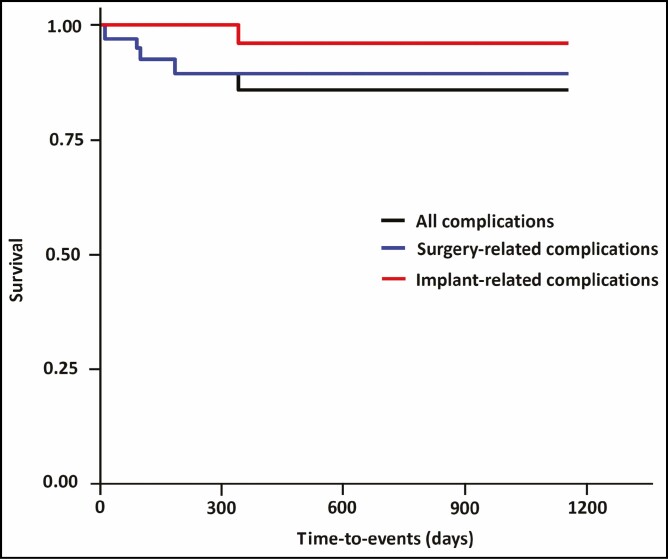
Kaplan-Meier cumulative survival. In our series, time-to-events were estimated at 266.81 ± 273.17 days.

**Figure 4.**
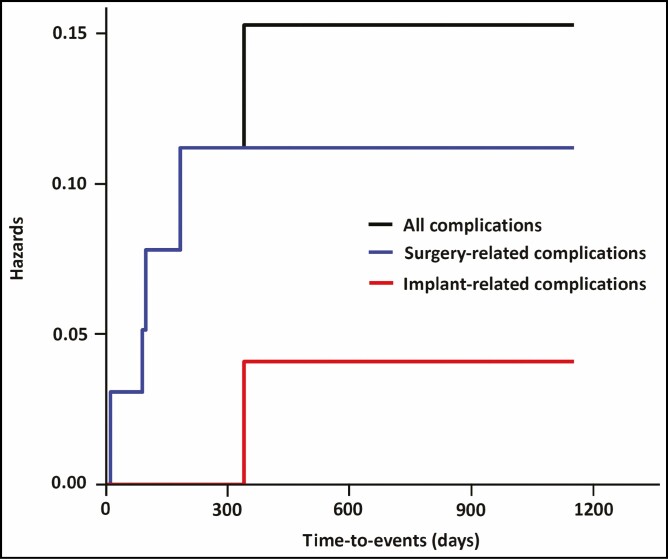
Kaplan-Meier cumulative hazards.

A total of 6 cases (8.7%) of postoperative complications occurred; these include 2 cases (2.9%) of early seroma, 1 case (1.4%) of CC, 2 cases (2.9%) of alterations in the shape, and 1 case (1.4%) of foreign body sensation (Table 3). In our series, time-to-events (TTEs) were estimated at 266.81 ± 273.17 days (Table 4). Moreover, cumulative complication-free survival is presented in Table 5. The corresponding Kaplan-Meier cumulative survival and hazards were plotted as a curve (Figures 3, 4).

**Table 3.** Postoperative Complications (n = 69)

Variable	Value	Treatment
Surgery-related complications		
Early seroma	2 (2.9%)	US-guided aspiration
CC	1 (1.4%)	Conservative therapy
Alterations in the shape	2 (2.9%)	Revision
Implant-related complications		
Foreign body sensation	1 (1.4%)	Explantation

Values are the number of the patients with percentage. CC, capsular contracture; US, ultrasound.

**Table 4.** Overall Complication-Free Survival

N	n	Censored value	TTEs (months)
69	6	63	266.81 ± 273.17

Values are mean ± standard error with 95% confidence interval. N, total number of cases; n, incidences of postoperative complications; TTEs, time-to-events.

**Table 5.** Cumulative Hazards

	FU	N	n	Survival rate
All complications	12	66	2	0.97 ± 0.0211 (0.929-1)
	90	49	1	0.95 ± 0.0285 (0.896-1)
	99	38	1	0.925 ± 0.0371 (0.855-1)
	184	30	1	0.894 ± 0.047 (0.807-0.991)
	340	25	1	0.858 ± 0.0571 (0.753-0.978)
Surgery-related complications	12	66	2	0.97 ± 0.0211 (0.929-1)
	90	49	1	0.95 ± 0.0285 (0.896-1)
	99	38	1	0.925 ± 0.0371 (0.855-1)
	184	30	1	0.894 ± 0.047 (0.807-0.991)
Implant-related complications	340	25	1	0.96 ± 0.0392 (0.886-1)

Values are mean ± standard error with 95% confidence intervals. FU, time points of follow-up; N, total number of cases; n, incidences of postoperative complications.

## DISCUSSION

Commercially available silicone gel-filled breast implants are equipped with diverse surface topographies, such as smooth, microtextured, and macrotextured surfaces.^7^ A novel system, the ISO 14607:2018, has been recently devised to classify diverse surface topographies of a device.^32,33^ That is, surface topography is analyzed using scanning electron microscopy and surface roughness (Ra).^33^ Thus, the surface of a breast implant is classified into smooth (Ra < 10 μm), microtextured (10 μm ≤ Ra ≤ 50 μm), or macrotextured19 (Ra > 50 μm).^34^ According to clinical studies, immune responses to foreign bodies as well as fibrosis vary depending on the surface topography.^35,36^ This is well illustrated in a causal relationship between BIA-ALCL and a textured device.^37^ The variability in the immune response to a breast implant depending on its surface topography has been based on hypothesis that surface texturing might inhibit the occurrence of CC by suppressing the fibrosis around the device.^38^ Moreover, a study also showed that a textured breast implant promoted inflammatory responses *as well as* chronic antigenic stimulation.^39^ Consequently, a persistent presence of inflammatory stimuli may lead to detrimental outcomes, such as pain, fluid collection, infection, or BIA-ALCL.^40^ Moreover, it is noteworthy that particulate debris are released from a textured surface and then engulfed by macrophages. This may eventually lead to persistent phagocytosis, increased synthesis of inflammatory cytokines, and increased lymphocyte proliferation.^41^ It has also been hypothesized that mechanical shear stress might trigger the occurrence of inflammatory responses, thus being possibly involved in the onset of double capsule and late seroma.^42^

A recent study analyzed immune responses by the surface topography of a silicone gel-filled breast implant in diverse types of commercially available products from the Establishment Labs Holdings Inc. (Motiva Ergonomix Round SilkSurface [microtextured] and VelvetSurface [microtextured]), the Mentor Worldwide LLC. (Santa Barbara, CA) (Mentor Smooth [smooth] and Mentor MemoryGel SILTEX [microtextured]), or the Allergan Inc. (MICROCELL [microtextured] and Natrelle INSPIRA BIOCELL [macroextured]). It also tested a hypothesis that the Motiva Ergonomix Round SilkSurface might alter the kinetics and characteristics of foreign body reactions. Thus, the long-term efficacy of the human-sized, commercial, and miniaturized Motiva Ergonomix Round SilkSurface in inhibiting the fibrosis in New Zealand White (NZW) rabbits (≤1 year) and C57BL/6 mice (≤6 months), respectively. Moreover, that study compared the profile of fibrosis between wild-type and T-cell-deficient C57BL/6 mice, thus showing that there was a significant reduction in the number of macrophages only in wild-type mice. Furthermore, the immune responses of the NZW rabbits and C57BL/6 mice were matched to those seen in human clinical specimens that were collected from the luminal surface of scar capsules formed for long period of time (7 months to 11 years) in patients receiving aesthetic or reconstructive augmentation mammaplasty.^34^

It has been described that a novel silicone gel-filled breast implant with a microtextured surface has emerged to overcome risks of both CC and BIA-ALCL. This is based on a hypothesis that surface texturing might be helpful for avoiding the parallel alignment of collagen fibers.^33,43^ But such hypothesis has been recently challenged. In addition, disadvantages of macrotextured devices, such as risks of late seroma, the formation of double capsule, and biofilm and BIA-ALCL, have also been revealed.^33^ The Motiva Ergonomix Round SilkSurface is equipped with a microtextured surface, thus known as an intermediate between smooth and conventional textured devices.^44^ According to Montemurro et al, there was a time-dependent decrease in the incidence of complications of the Motiva Ergonomix Round SilkSurface, which may be attributable to a learning curve.^44^ But these authors also admitted that a refinement in dissection technique for very tight implant pockets was needed to minimize the inferior and lateral migration. Additionally, they also reported that good soft tissue elasticity and lower volume (<350 cc) were essential factors that may lead to acceptable clinical outcomes of an implant-based augmentation mammaplasty using the Motiva Ergonomix Round SilkSurface.^44^

We have efficiently combined the use of HRUS with an implant-based augmentation mammaplasty using the Motiva Ergonomix Round SilkSurface, thus making an effort to detect its complications at the earliest opportunities possible. We found that there were a total of 6 cases (8.7%) of postoperative complications during a 3-year period. A relatively higher incidence of early seroma (2.9%) remains problematic; it was shown to occur at a rate of 1.32% (4/76) during a 1-year period.^13^ The formation of early seroma is defined as the accumulation of periprosthetic fluid within the first postoperative year.^45^ We efficiently manage patients with early seroma using HRUS-guided aspiration.

To date, previous studies have shown that incidences of CC range between 0.0% and 2.10% in a cohort of patients receiving the Motiva Ergonomix Round SilkSurface.^13,19,22,23,46^ We found that CC occurred at an incidence of 1.4%; it was within the previous range and higher as compared with that reported during a 1-year period (0.0%).^13^

Limitations of the current study are as follows: First, we included a small series of the patients under the retrospective design. Second, we conducted the current study in a cohort of the patients who visited a single local clinic in Seoul, Korea. Therefore, the possibility of selection bias could not be completely ruled out. Third, we described methods for assessing CC based on TC within the scope of empirical experience. We suggest that a prospective multi-center study with a larger cohort is necessary to put these findings into context and make them replicable.

## CONCLUSIONS

According to Deva et al, the breast implant industry has been prone to crisis.^47^ More specifically, K Groth and Graf further classified the breast implant crisis into the first crisis (Dow Corning), the second crisis (Poly Implant Prothèse [PIP]), and the third crisis (BIA-ALCL).^48^

Surgeons, patients, and the breast implant industry in Korea have recently experienced a crisis from BIA-ALCL and the first Korean case of a medical device fraud.^12,14,15^ Between 2019 and 2020 (August 16 and December 24, 2019, and October 5, 2020), 3 cases of BIA-ALCL occurred in Korea.^49^ This led to a ban of textured breast implants mandated by the KMFDS on August 29, 2019. Since then, no textured devices have been permitted until present. Later, on November 13, 2020, mandatory recall of the BellaGel breast implants, including the BellaGel SmoothFine, was initiated by the KMFDS. According to the news media, the manufacturer, the HansBiomed Co. Ltd., was investigated by the Korean police for using unapproved substances, such as 7-9700 and Q7-4850, and deliberately modifying the shell structure from 5 to 4 layers during the manufacturing process.^12,14^ Kim reported that the manufacturer was previously involved in the PIP fraud in Europe.^12,14^ Currently in Korea, only smooth and microtextured breast implants are commercially available; these include the Motiva Ergonomi Round SilkSurface (Establishment Labs Holdings Inc.), the SEBBIN Integrity, Sublimity and Purity (Groupe Sebbin SAS, Boissy-l′Aillerie, France), the Eurosilicone Round Collection (GC Aesthetics PLC, Apt Cedex, France), the Mentor MemoryGel Xtra (Mentor Worldwide LLC.), and the Natrelle INSPIRA (Allergan Inc.). According to Song et al, the Motiva Ergonomi Round SilkSurface might be a device of choice for Korean females who have faced a crisis from BIA-ALCL and the first Korean case of a medical device fraud.^20^

In conclusion, we describe our preliminary short-term safety outcomes of an implant-based augmentation mammaplasty using the Motiva Ergonomix Round SilkSurface in Korean females. But this deserves further large-scale studies with long periods of follow-up.
